# Blood KL-6 predicts prognosis in primary Sjögren’s syndrome-associated interstitial lung disease

**DOI:** 10.1038/s41598-022-09283-w

**Published:** 2022-03-29

**Authors:** Yun Jae Kim, Jooae Choe, Su-Jin Moon, Jin Woo Song

**Affiliations:** 1 University of Ulsan College of Medicine, Seoul, Republic of Korea; 2 Department of Radiology and Research Institute of Radiology, University of Ulsan College of Medicine, Asan Medical Center, Seoul, Republic of Korea; 3 Department of Pulmonary and Critical Care Medicine, Asan Medical Center, University of Ulsan College of Medicine, 88 Olympic-ro 43-gil, Songpa-gu, Seoul, 05505 Republic of Korea

**Keywords:** SjÃ¶gren's disease, Prognostic markers

## Abstract

Interstitial lung disease associated with primary Sjögren’s syndrome (SJS-ILD) has a variable clinical course. We aimed to investigate the role of blood biomarkers in predicting prognosis for SJS-ILD. Clinical data of 46 SJS-ILD patients were retrospectively reviewed. Plasma biomarker levels, including Krebs von den Lungen-6 (KL-6), CC chemokine ligand 18 (CCL18), chitinase-3-like-1 (YKL-40), interleukin-4 receptor alpha (IL-4Ra), and matrix metalloproteinase-7 (MMP-7) were measured using the multiplex Luminex assays (R&D Systems, Minneapolis, USA). The median follow-up period was 69.0 months. The mean age of the patients was 59.4 years; 17.4% were men. The KL-6 level was significantly higher in non-survivors (n = 12; 119.6 vs. 59.5 pg/mL, *P* = 0.037) than survivors (n = 34), while the levels of the other biomarkers did not differ. Receiver operating characteristic analysis indicated that KL-6 shows the best performance for predicting survival (area under the curve = 0.705, *P* = 0.037; best cut-off value = 53.5 pg/mL). Multivariable Cox analysis that was adjusted by age and diffusing capacity for carbon monoxide suggested a high KL-6 level (> 53.5 pg/mL) as an independent prognostic factor for survival (hazard ratio = 5.939, 95% confidence interval 1.312–26.881, *P* = 0.021). Our results suggest that blood KL-6 might be a useful in predicting the prognosis for patients with SJS-ILD.

## Introduction

Primary Sjögren syndrome (SJS) is a chronic systemic inflammatory disorder that is characterized by diminished function of the lacrimal and salivary glands^1–3^. Lung involvement is a common extraglandular complication; the most frequent lung complication, interstitial lung disease (ILD), has a reported prevalence of 10–20%^4–6^. ILD has been reported to be associated with increased mortality in patients with SJS^7,8^. Although SJS-ILD generally follows a mild and self-limited course, it can exhibit a more severe and progressive course in some patients^9^. Therefore, it is important to find predicting factors that could differentiate those patients who will have the progressive disease from those that are expected to experience slow or stable disease, in order to provide appropriate intervention. Prognostic factors for patients with SJS-ILD, such as the baseline partial pressure of carbon dioxide and oxygen, forced vital capacity (FVC), the extent of reticular abnormality on high resolution computed tomography (HRCT), and the severity of fibroblastic foci, or the presence of microscopic honeycomb in a surgical lung biopsy have been reported previously as predicting factors for mortality in SJS-ILD^10–12^. However, the utility of these variables can be limited by insufficient effort on the part of the patient, reader variability in the interpretation of images, or invasiveness.

On the contrary, blood biomarkers are relatively easy to test, independent of patient effort or reader ability. Biomarkers for idiopathic pulmonary fibrosis (IPF) or connective tissue disease (CTD)-ILD have mainly been investigated using proteins that are associated with epithelial damage, matrix turnover, or inflammation^13^. The most-studied epithelial-specific biomarker in ILD is Krebs von den Lungen-6 (KL-6)^10,14,15^. KL-6 is a mucin-like glycoprotein that is of high molecular weight and is classified as a human MUC1 mucin protein. It is secreted by type II alveolar pneumocytes and bronchial epithelial cells in response to cellular damage^16^ and is therefore frequently considered an indicator for pulmonary damage and has been studied as a biomarker for disease activity in ILD^17,18^. The cytokines and enzymes that have been reported to be associated with the clinical course of IPF or CTD-ILDs include CC chemokine ligand 18 (CCL18), chitinase-3-like protein 1 (YKL 40), and interleukin-4 receptor alpha (IL-4Ra)^12,14,15,19–22^. CCL18 is a chemokine that is secreted mainly by alveolar macrophages (M2 phenotype)^23^ and is known to play an important role in the immune-mediated lung fibrosis processes in IPF^24^, and YKL 40 is a glycoprotein that is primarily secreted by macrophages, neutrophils, and certain types of local epithelial cells and is known to have a role in inflammation and tissue remolding^25^. A recent meta-analysis study revealed that serum YKL-40 is correlated with lung function and can therefore be used as a predictive biomarker for survival with IPF and CTD-ILD^26^. IL-4R mainly promotes the proliferation of T cells and B cells^22^, and can also stimulate the proliferation, differentiation, and activation of several other cell types including fibroblasts while also increasing the recruitment of inflammatory cells^22^. The extracellular matrix markers in ILD include matrix metalloproteinase-7 (MMP-7)^27–29^. The MMPs are a group of proteins that contribute to the activation of pro-fibrotic molecules, crucially contributing to tissue remodeling and fibrosis^30,31^. Of these, MMP-7 is reported to be associated with the severity and prognosis of IPF, suggesting its role in the fibrotic process^27,28^. However, the role of blood biomarkers in predicting prognosis in SJS-ILD is not well defined, and no study has yet compared the performance of various biomarkers in predicting SJS-ILD prognosis. Our study therefore aimed to compare the prognostic value of the different blood biomarkers to find the best biomarker for use in patients with SJS-ILD.

## Methods

### Study population

A total of 62 patients diagnosed with primary SJS-ILD (biopsy-proven cases, n = 16) between January 2000 and December 2016 at Asan Medical Center, Seoul, Republic of Korea, were screened in this study. Of these, only subjects with available blood samples were finally included in this study (n = 46). The patients excluded from the cohort showed lower C-reactive protein level than those included (see Supplementary Table S1). All patients met the diagnostic criteria of the American College of Rheumatology and the European League Against Rheumatism (EULAR), and the presence of ILD was confirmed by HRCT images^32^. The study was approved by the Institutional Review Board of Asan Medical Center (2018-1115), and written informed consent for the use of the blood samples for clinical research was obtained from all patients. All methods were performed in accordance with the relevant guidelines and regulations.

### Clinical data

The clinical and survival data of all patients were retrospectively collected from medical records, and/or the records of the National Health Insurance Service of Korea. To measure disease activity in patients with primary SJS, EULAR Sjögren’s Syndrome Disease Activity Index (ESSDAI) scores at the time of ILD diagnosis was calculated^33^. Spirometry, total lung capacity (TLC) and the diffusing capacity of the lung for carbon monoxide (DL_CO_) were measured according to the recommendations from the American Thoracic Society (ATS)/European Respiratory Society (ERS). The results were presented as percentages of the normal predicted values^34–36^. A six-minute walk test (6MWT) was performed according to ERS/ATS recommendations^37^, and bronchoalveolar lavage (BAL) was performed according to the ATS guildelines^38^. Data from follow-up assessments at 3–6-month intervals or from hospitalization events were reviewed to determine the development of acute exacerbation (AE). AE was defined according to the criteria suggested by Collard et al.^39^, which is worsening dyspnea within 30 days with new bilateral lung infiltration with no evidence of infection or other alternative causes for the dyspnea (e.g., pulmonary embolism or heart failure).

### Measurement of blood biomarkers

Blood samples were obtained at the time of diagnosis by venipuncture and immediately centrifuged. The separated plasma samples were then stored at − 80 °C until biomarker measurement. The plasma levels of KL-6, CCL18, YKL-40, IL-4Ra, and MMP-7 were measured using the multiplex Luminex assays (R&D Systems, Minneapolis, USA) in accordance with the manufacturer’s instructions.

### HRCT evaluation

HRCT scans were obtained in accordance with standard protocols and reviewed by a radiologist (J.C.) who was blinded to the clinical information. The HRCT patterns were categorized into usual interstitial pneumonia (UIP), probable UIP, indeterminate for UIP, or an alternative diagnosis based on the 2018 Fleischner Society guidelines^3^. A UIP pattern was defined by a subpleural and basal predominance of reticular abnormalities, honeycombing with or without traction bronchiectasis, and the absence of inconsistent findings with a UIP pattern such as extensive ground-glass opacities (GGO), micro-nodules, discrete cysts, or segmental/lobar consolidations^40^.

### Statistical analysis

All values were expressed as mean ± standard deviation for continuous variables or percentages for categorical variables. The Student’s *t*-test or Mann–Whitney U test were used for the continuous data, while the Pearson’s chi-squared or Fisher’s exact test were utilized to analyze the categorical data. ROC curve analysis was performed to evaluate the optimal cut-off value of blood biomarkers for predicting survival. The risk factors for mortality were evaluated using a Cox proportional hazard model. Due to the limited number of death events, among variables with *P* < 0.1, age, and DL_CO_, which were previously prognostic factors in SJS-ILD^41,42^. were used as adjustment variables for the multivariable Cox analysis. Survival was evaluated using Kaplan–Meier survival analysis and the log-rank test. Survival time was calculated from the date of ILD diagnosis to death or censoring, which took place on December 31, 2016 and included all patients who were still alive on this date. Spearman’s rank correlation coefficients were performed to evaluate the correlation between blood KL-6 levels and lung function or exercise capacity. All *P*-values were two-tailed with statistical significance set at a *P* value of < 0.05, and all statistical analyses were performed using SPSS Statistics, Version 24.0. (IBM Corp., Armonk, NY, USA).

## Results

### Baseline characteristics

The mean age of the 46 patients with primary SJS-ILD was 59.4 years and 17.4% were males (Table 1). The median follow-up period was 69.0 months (interquartile range [IQR], 23.0–101.8 months), with 12 patients (26.1%) dying during follow-up. The major cause of death was underlying ILD progression (66.7%), followed by pneumonia, tuberculosis, heart failure, and unknown cause (8.3% each) (Supplementary Table S2).

**Table 1:** Comparison between the baseline characteristics of non-survivors and survivors in patients with SJS-ILD.

Characteristics	Total	Non-survivors	Survivors	*P* value
Patient number	46	12	34	
Age (years)	59.4 ± 10.6	64.8 ± 2.8	57.5 ± 10.4	0.052
Male	8 (17.4)	2 (16.7)	6 (17.6)	1.000
Smoking history	12 (26.1)	6 (50.0)	6 (17.6)	0.052
ESSDAI	12.7 ± 4.1	14.2 ± 3.8	12.1 ± 4.0	0.136
ANA, positive (> 1:40)	34 (73.9)	8 (66.7)	26 (76.5)	0.703
Anti SS-A/Ro, positive	33 (71.7)	5 (41.7)	28 (82.4)	0.021
Anti-SS-B/La, positive	16 (34.8)	2 (16.7)	14 (41.2)	0.170
C-reactive protein (mg/dL)	1.4 ± 2.9	2.9 ± 5.0	0.8 ± 1.2	0.165
FVC, predicted %	67.2 ± 13.7	61.3 ± 13.4	69.2 ± 13.4	0.115
DL_CO_, predicted %	57.7 ± 18.4	50.6 ± 16.7	60.2 ± 18.5	0.152
TLC, predicted %	70.7 ± 13.7	63.0 ± 13.9	73.1 ± 12.9	0.045
6MWD (m)	425.1 ± 107.1	355.6 ± 43.9	442.4 ± 18.8	0.063
6MWT the lowest SpO_2_, %	91.2 ± 4.3	89.1 ± 4.5	91.7 ± 4.2	0.147
BAL neutrophil (%)	12.4 ± 18.2	31.7 ± 41.4	8.3 ± 6.2	0.568
BAL lymphocyte (%)	27.2 ± 15.8	9.0 ± 8.9	30.3 ± 14.7	0.026
UIP pattern on HRCT	19 (41.3)	10 (83.3)	9 (26.5)	0.001
Treatment with steroid ± IM^a^	39 (84.8)	9 (75.0)	30 (88.2)	0.255
Initial dosage of steroid^b^	31.8 ± 12.2	33.3 ± 10.9	31.39 ± 12.7	0.681
Treatment duration (months)^c^	15 (6–33)	8 (3.5–31)	17.5 (6.8–33.5)	0.205

Data are presented as mean ± standard deviation or number (%), unless otherwise indicated.

*6MWD* six-minute walk test distance, *6MWT the lowest SpO2* lowest oxygen saturation during the six-minute walking test, *ANA* anti-nuclear antibody, *BAL* bronchoalveolar lavage, *DL*_*CO*_ diffusing capacity of the lung for carbon monoxide, *ESSDAI* European Alliance of Associations for Rheumatology Sjögren’s Syndrome Disease Activity Index, *FVC* forced vital capacity, *HRCT* high resolution computed tomography, *ILD* interstitial lung disease, *IM* immunosuppressants, *SJS* Sjögren syndrome, *TLC* total lung capacity, *UIP* usual interstitial pneumonia.

^a^The immunosuppressants included azathioprine (n = 20), cyclosporine (n = 16), mycophenolate mofetil (n = 21), and cyclophosphamide (n = 6).

^b^Prednisolone equivalent dose.

^c^Median (interquartile range).

The non-survivors had lower TLC and lymphocyte levels in their BAL fluid, and showed positive anti SS-A/Ro and a UIP pattern on the HRCT more frequently than the survivors (Table 1). Total of 39 patients (84.8%) received steroid and/or immunosuppressants (median treatment duration: 15 months [interquartile range: 6–33 months]. However, there was no difference between non-survivors and survivors in terms of the number of patients treated, the initial steroid dose, and the duration of the treatment given.

### Comparison of blood biomarkers

**Figure 1:**
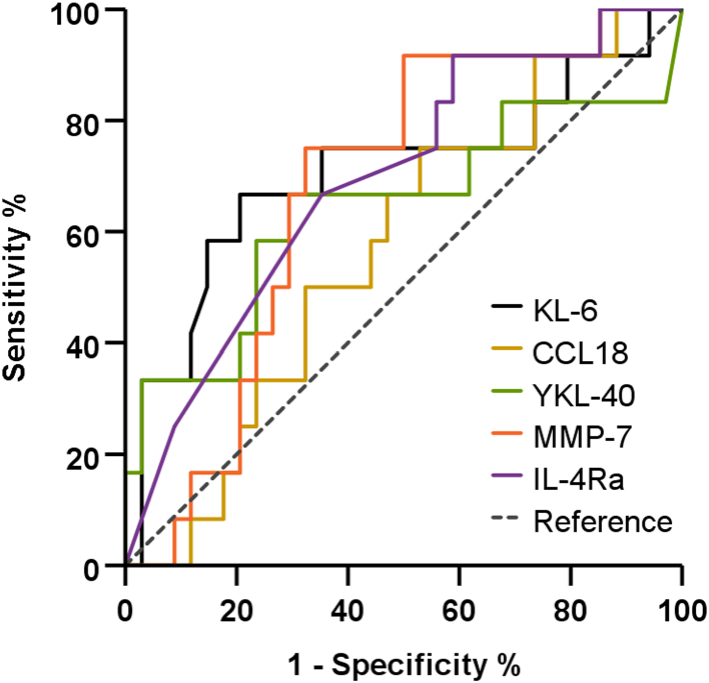
Comparison of the ROC curves of biomarkers for 10-year mortality in patients with SJS-ILD. KL-6 (AUC = 0.750, 95% CI 0.509–0.901; *P* = 0.037), CCL18 (AUC = 0.569, 95% CI 0.391–0.746, *P* = 0.484), YKL-40 (AUC = 0.642, 95% CI 0.431–0.854, *P* = 0.147), IL-4Ra (AUC = 0.692, 95% CI 0.539–0.820, *P* = 0.053), MMP-7 (AUC = 0.676, 95% CI 0.513–0.840, *P* = 0.072). *AUC* area under the curve, *CCL18* CC chemokine ligand 18, *CI* confidence interval, *ILD* interstitial lung disease, *IL-4Ra* interleukin-4 receptor alpha, *KL-6* Krebs von den Lungen-6, *MMP-7* matrix metalloproteinase-7, *SJS* Sjögren syndrome, *YKL-40* chitinase-3-like-1.

The level of KL-6 was significantly elevated in the non-survivors (119.6 vs 59.5 pg/mL, *P* = 0.037) as compared to the survivors (Table 2). However, no significant differences were observed between the two groups for the other biomarker levels. KL-6 was the most significant predictor of mortality (area under a curve [AUC] = 0.705, 95% confidence interval [CI] 0.509–0.901, *P* = 0.037) using ROC analysis for 10-year survival, and the optimal cut-off value was 53.5 pg/mL (sensitivity = 66.7%, specificity = 79.4%) (Fig. 1). The rest of the biomarkers were lesser predictive of survival than KL-6 (CCL18 [AUC = 0.569, 95% CI 0.391–0.746, *P* = 0.484], YKL-40 [AUC = 0.642, 95% CI 0.431–0.854, *P* = 0.147], IL-4Ra [AUC = 0.690, 95% CI 0.518–0.862, *P* = 0.053], and MMP-7 [AUC = 0.676, 95% CI 0.513–0.840, *P* = 0.072]).

**Table 2:** Baseline levels of blood biomarkers in non-survivors and survivors for patients with SJS-ILD.

Characteristics	Total	Non-survivors	Survivors	*P* value
KL-6 (pg/mL)	75.2 ± 116.0	119.6 ± 124.0	59.5 ± 110.7	0.037
CCL18 (ng/mL)	63.8 ± 31.6	66.1 ± 23.6	62.9 ± 34.3	0.484
YKL-40 (ng/mL)	114.1 ± 131.3	187.4 ± 206.9	88.2 ± 81.2	0.147
IL-4Ra (ng/mL)	2.2 ± 4.3	0.5 ± 1.6	2.8 ± 4.8	0.053
MMP-7 (ng/mL)	2.6 ± 2.3	3.6 ± 1.7	2.2 ± 2.4	0.072

Data are presented as mean ± standard deviation or number (%), unless otherwise indicated.

*CCL18* CC chemokine ligand 18, *IL-4Ra* interleukin-4 receptor alpha, *ILD* interstitial lung disease, *KL-6* Krebs von den Lungen-6, *MMP-7* matrix metalloproteinase-7, *SJS* Sjögren syndrome, *YKL-40* chitinase-3-like-1.

The unadjusted Cox proportional hazards model showed that age, smoking status, higher C-reactive protein levels, lower DL_CO_ and TLC, a shorter six-minute walk test distance (6MWD), a UIP pattern on the HRCT, and a higher level of KL-6 (> 53.5 pg/mL) were significantly associated with 10-year mortality (Table 3). In the multivariable analysis adjusted by age and DL_CO_, a high KL-6 level (> 53.5 pg/mL) was independently associated with a poor prognosis (hazard ratio [HR] = 5.939, 95% CI 1.312–26.881, *P* = 0.021) (Table 4). However, no association was observed between any of the other biomarkers and mortality in patients with SJS-ILD.

**Table 3:** Risk factors for mortality in patients with SJS-ILD assessed using a unadjusted Cox proportional hazards model.

Variables	HR	95% CI	P value
Age (years)	1.081	1.015–1.152	0.015
Male sex	1.299	0.282–5.984	0.737
Smoking history	4.687	1.472–14.924	0.009
ESSDAI	1.128	0.977–1.302	0.099
ANA, positive (> 1:40)	0.648	0.195–2.161	0.481
Anti SS-A/Ro, positive	0.335	0.106–1.058	0.062
Anti-SS-B/La, positive	0.339	0.074–1.549	0.163
C-reactive protein (mg/dL)	1.338	1.119–1.600	0.001
FVC, predicted %	0.968	0.928–1.010	0.130
DL_CO_, predicted %	0.970	0.936–1.005	0.093
TLC, predicted %	0.958	0.917–1.000	0.052
6MWD (m)	0.990	0.983–0.997	0.008
6MWT, the lowest SpO_2_	0.908	0.769–1.072	0.255
UIP pattern on HRCT	11.034	2.406–50.612	0.002
KL-6 (> 53.5 pg/mL)	6.004	1.778–20.270	0.004
CCL18 (> 54.0 ng/mL)	2.042	0.552–7.560	0.285
YKL-40 (> 85.7 ng/mL)	2.577	0.775–8.569	0.122
IL-4Ra (≤ 0.2 ng/mL)	2.344	0.293–18.767	0.422
MMP-7 (> 2.9 ng/mL)	2.455	0.659–9.153	0.181

*6MWD* six-minute walk test distance, *6MWT the lowest SpO2* lowest oxygen saturation during the six-minute walking test, *CCL18* CC chemokine ligand 18, *DL*_*CO*_ diffusing capacity of the lung for carbon monoxide, *ESSDAI* European Alliance of Associations for Rheumatology Sjögren’s Syndrome Disease Activity Index, *FVC* forced vital capacity, *HR* hazard ratio, *HRCT* high resolution computed tomography, *IL-4Ra* interleukin-4 receptor alpha, *ILD* interstitial lung disease, *KL-6* Krebs von den Lungen-6, *MMP-7* matrix metalloproteinase-7, *SJS* Sjögren’s syndrome, *TLC* total lung capacity, *UIP* usual interstitial pneumonia, *YKL-40* chitinase-3-like-1.

**Table 4:** Risk factors for mortality in patients with SJS-ILD assessed using a multivariable Cox proportional hazards model.

Variables^a^	HR	95% CI	*P* value
KL-6 (> 53.5 pg/mL)	5.939	1.312–26.881	0.021
CCL18 (> 54.0 ng/mL)	2.073	0.408–10.537	0.379
YKL-40 (> 85.7 ng/mL)	1.407	0.392–5.059	0.601
IL-4Ra (≤ 0.2 ng/mL)	2.744	0.335–22.488	0.347
MMP-7 (> 2.9 ng/mL)	1.613	0.404–6.440	0.499

*CCL18* CC chemokine ligand 18, *CI* confidence interval, *HR* hazard ratio, *IL-4Ra* interleukin-4 receptor alpha, *ILD* interstitial lung disease, *KL-6* Krebs von den Lungen-6, *MMP-7* matrix metalloproteinase-7, *SJS* Sjögren’s syndrome, *YKL-40* chitinase-3-like-1.

^a^Adjusted by age and DL_CO_.

### Survival according to KL-6 levels

**Figure 2:**
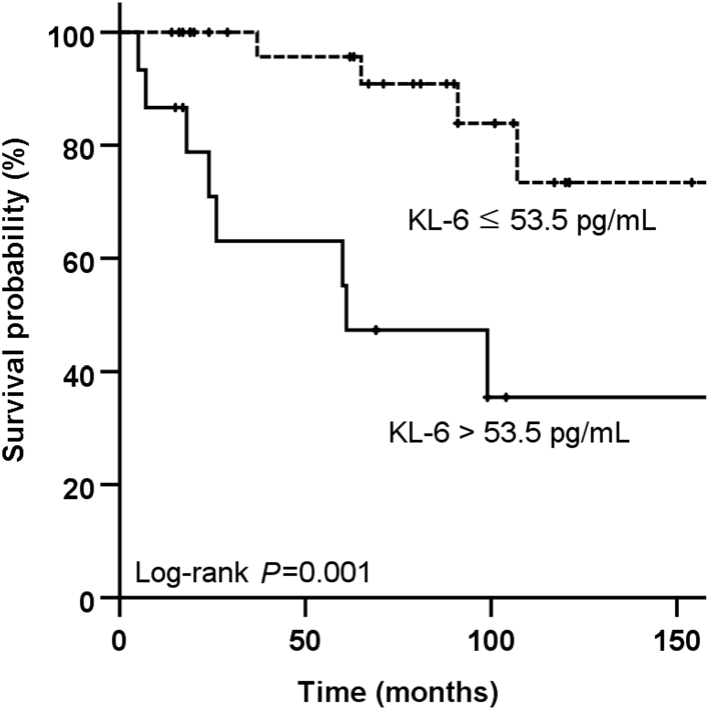
Comparison of the Kaplan–Meier survival curves according to KL-6 levels in patients with SJS-ILD. Vertical axis represents survival probability (%); horizontal axis represents time (months) after diagnosis. Black line indicates high KL-6 group (> 53.5 pg/mL) and dotted line indicates low KL-6 group (≤ 53.5 pg/mL). Vertical bar indicates a censored case. *ILD* interstitial lung disease, *KL-6* Krebs von den Lungen-6, *SJS* Sjögren syndrome.

Classification of the patients in accordance with the baseline level for KL-6 demonstrated lower lung functions (FVC, DL_CO_, TLC) and poorer exercise capacities (shorter walking distance and lower the minimum oxygen saturation on 6MWT) in the high KL-6 group (> 53.5 pg/mL, n = 15) than the low KL-6 group (≤ 53.5 pg/mL, n = 31) (Table 5). Patients with high KL-6 levels (with a mean follow-up period of 61.9 months) also showed more frequent AE (4 patients [27%] vs 1 patient [3%], *P* = 0.017) than the low KL-6 group (81.5 months; *P* = 0.288) and were less likely to survive (5-year survival: 64% vs 96%; 10-year survival: 30.0% and 75.0%; *P* = 0.001) than those with low KL-6 levels. (Fig. 2).

**Table 5:** Comparison between the clinical characteristics for high and low KL-6 groups in patients with SJS-ILD.

	High KL-6 (> 53.5 pg/mL)	Low KL-6 (≤ 53.5 pg/mL)	*P* value
Patients number	15	31	
Age	61.3 ± 10.7	58.5 ± 10.6	0.496
Male	1 (7)	7 (23)	0.243
Smoking history	6 (40)	6 (19.4)	0.165
ESSDAI	13.9 ± 3.9	12.1 ± 4.1	0.162
ANA, positive (> 1:40)	10 (66.7)	24 (77.4)	0.488
Anti SS-A/Ro, positive	11 (73.3)	22 (71.0)	1.000
Anti-SS-B/La, positive	3 (20)	13 (41.9)	0.195
C-reactive protein (mg/dL)	2.4 ± 4.5	0.8 ± 1.3	0.213
FVC, predicted %	56.3 ± 12.7	72.4 ± 10.9	0.000
DL_CO_, predicted %	45.8 ± 15.9	63.5 ± 16.8	0.002
TLC, predicted %	62.4 ± 13.6	75.0 ± 11.7	0.004
6MWD (m)	355.3 ± 83.7	461.4 ± 101.0	0.004
6MWT the lowest SpO_2_, %	88.2 ± 3.5	92.8 ± 4.0	0.002
BAL neutrophils (%)	25.8 ± 30.4	6.8 ± 5.3	0.237
BAL lymphocyte (%)	30.5 ± 22.3	25.9 ± 13.1	0.563
UIP pattern on HRCT	7 (46.7)	12 (38.7)	0.607
Treatment with steroid ± IM^a^	14 (93.3)	24 (77.4)	0.243
Initial dosage of steroid^b^	32.1 ± 15.5	31.7 ± 10.2	0.919
Treatment duration (months)^c^	11 (6–31)	12 (3.8–47.3)	0.616

Data are presented as mean ± standard deviation or number (%), unless otherwise indicated.

^a^The immunosuppressants included azathioprine (n = 20), cyclosporine (n = 16), mycophenolate mofetil (n = 21), and cyclophosphamide (n = 6).

^b^Prednisolone equivalent dose.

^c^Median (interquartile range).

*6MWD* six-minute walk test distance, *6MWT the lowest SpO2* lowest oxygen saturation during the six-minute walking test, *ANA* anti-nuclear antibody, *BAL* bronchoalveolar lavage, *DL*_*CO*_ diffusing capacity of the lung for carbon monoxide, *ESSDAI* European Alliance of Associations for Rheumatology Sjögren’s Syndrome Disease Activity Index, *FVC* forced vital capacity, *HRCT* high resolution computed tomography, *ILD* interstitial lung disease, *KL-6* Krebs von den Lungen-6, *SJS* Sjögren syndrome, *TLC* total lung capacity, *UIP* usual interstitial pneumonia.

### Correlation between KL-6 and physiological parameters

**Figure 3:**
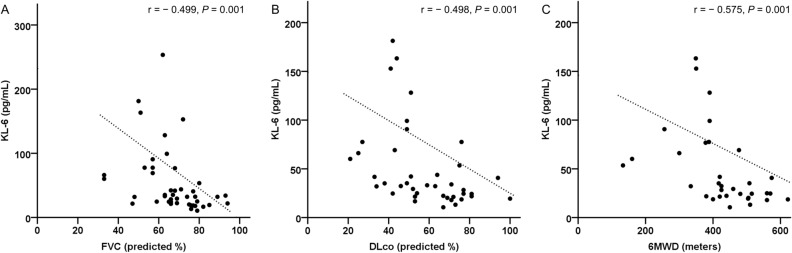
Correlation between KL-6 levels and physiological parameters in patients with SJS-ILD. Serum KL-6 showed negative correlation with (**A**) FVC (r, − 0.499; *P* = 0.001), (**B**) DL_CO_ (r, − 0.498, *P* = 0.001), (**C**) 6MWD (r, − 0.575; *P* = 0.001). Spearman’s correlation coefficients were used to analyze the linear relationship between the variables. *6MWD* six-minute walk test distance, *DL*_*CO*_ diffusing capacity of the lung for carbon monoxide, *FVC* forced vital capacity, *ILD* interstitial lung disease, *KL-6* Krebs von den Lungen-6, *SJS* Sjögren syndrome.

Significant negative correlations were observed between the KL-6 levels and FVC (r =  − 0.499, *P* = 0.001), DL_CO_ (r =  − 0.498, *P* = 0.001), and 6MWD (r =  − 0.575, *P* = 0.001) (Fig. 3). However, no significant correlation was observed between the other biomarkers and lung function or exercise capacity.

## Discussion

In this study, the prediction of survival using various biomarkers was evaluated for patients with primary SJS-ILD. We focused on evaluating whether biomarkers that were previously studied in other CTD-ILDs are useful in patients with SJS-ILD in terms of the severity and prognosis for ILD. KL-6 was found to be superior to CCL18, YKL-40, IL-4Ra, and MMP-7 in predicting the prognosis of patients with SJS-ILD. High KL-6 levels were independently associated with an increased risk of 10-year mortality in patients with SJS-ILD when adjusted by age and DL_CO_. Patients with high KL-6 levels showed poorer lung function, with more frequent AE and death than those with low KL-6 levels. Moreover, KL-6 levels were found to correlate with the severity of the disease in patients with SJS-ILD.

KL-6 showed the best performance in predicting 10-year mortality as compared to the other biomarkers. A high KL-6 level (> 53.5 pg/mL) was found to be an independent risk factor for mortality after adjustment for age and DL_CO_ when using multivariable Cox analysis. These findings are consistent with those in previous reports^12,19^. Kamiya et al. studied 99 patients with SJS-ILD (with a median follow-up period of 5.97 years) and reported that a higher level of KL-6 (> 800 U/mL) was associated with poor survival (HR = 2.91, 95% CI 1.04–8.10, *P* = 0.04) using multivariable Cox analysis adjusted by age and gender^12^. Kim et al. also showed that a high KL-6 level (≥ 640 U/mL) was an independent prognostic factor for survival (HR = 3.235, 95% CI 1.394–7.510, *P* = 0.006) in 158 patients with rheumatoid arthritis (RA)-ILD^1919^. These findings suggest that KL-6 might be useful for predicting the clinical outcomes of CTD-ILD, including SJS-ILD. In addition to identifying the usefulness of KL-6 for predicting the prognosis of patients with SJS-ILD, our study revealed that KL-6 showed better performance for predicting survival compared to other biomarkers.

In this study, patients with high KL-6 levels experienced AE more frequently, and KL-6 levels were negatively correlated with lung function and exercise capacity, suggesting the value of KL-6 for predicting AE and disease severity. Previous reports support our findings^17,43^. Ohshimo et al. studied 77 patients with IPF and reported that a high KL-6 level (≥ 1300 U/mL) was an independent risk factor for the development of AE (HR = 11.8, 95% CI 1.43–97.8, *P* = 0.022) when adjusted for age, sex, smoking history, and treatment^43^. They also showed that baseline KL-6 levels were significantly higher in patients who experienced AE than those who did not (2528 ± 1645 U/mL vs. 1584 ± 1000 U/mL, *P* < 0.0001)^43^. Lee et al. studied 165 patients with CTD-ILD (41 RA, 53 systemic sclerosis, 56 inflammatory myopathy, 15 systemic lupus erythematosus or SJS), and also showed that the semiquantitative grades of ILD on the HRCT (grade 1, 0–25%; grade 2, 26–50%; grade 3, 51–75%; grade 4, 76–100%) were significantly proportional to serum KL-6 levels, from which grades could be successfully differentiated (grades 1 vs. 2, *P* = 0.022; grades 2 vs. 3, *P* < 0.001; grades 3 vs. 4, *P* = 0.002)^17^. They also showed that serum KL-6 level had a moderate negative correlation with both FVC% (r =  − 0.399, *P* < 0.001) and DL_CO_% (r =  − 0.578, *P* < 0.001)^17^.

In our study, CCL18, YKL-40, IL-4Ra, and MMP-7 were not found to be associated with the prognosis or severity of ILD. However, previous studies have reported contradictory findings^20–22,29^, with several reporting these biomarkers useful in the prognosis of CTD-ILD. Tiev et al. studied 83 patients with systemic sclerosis-ILD, and reported that a high baseline CCL18 level (> 187 mg/mL) was a predictive factor (HR = 5.36, 95% CI 2.44–11.75, *P* = 0.001) for worsening of the subsequent disease (with a decrease of > 10% predicted in FVC or TLC) within 2 years using multivariable Cox analysis^20^. Hozumi et al. also demonstrated a correlation between serum YKL-40 levels and a lower arterial oxygen pressure (r =  − 0.40, *P* < 0.001) in 72 patients with polymyositis/dermatomyositis-ILD, and independent association with poor prognosis under multivariable Cox analysis when adjusted by the anti-aminoacyl tRNA synthetase status (per 10 ng/ml increase, HR = 1.15, 95% CI 1.04–1.28, *P* < 0.01) or the anti-CADM-140/melanoma differentiation-associated gene 5 antibody status (per 10 ng/mL increase, HR = 1.15, 95% CI 1.04–1.29, *P* < 0.01)^21^. Moreover, Nakatsuka et al. studied 52 patients with polymyositis/dermatomyositis-ILD, and showed that higher levels of serum MMP-7 were associated with 6-month mortality (odds ratio [OR] = 1.57, 95% CI 1.01–2.45, *P* = 0.046) using univariate logistic regression analysis, and that high serum MMP-7 (> 5.08 ng/mL) was associated with a worse prognosis (OR = 14.60, 95% CI 1.11–192.00, *P* = 0.027) when using multivariable logistic regression analysis adjusted by hypoxia and serum ferritin levels^29^. Due to these inconsistent results, the prognostic value of the above markers is not clear for SJS-ILD. Further verification on a larger scale is required for clinical use in patients with SJS-ILD.

In this study, positive anti-SSA was more frequent in survivors but was not associated with prognosis in the unadjusted Cox analysis. Moreover, there was no difference in the frequency of positive autoantibodies such as ANA and anti-SSB between the non-survivors and survivors. The previous study support our findings; Gao et al., in 178 patients with SJS-ILD, reported that the frequency of positive ANA (65.2 vs 75.8%, *P* = 0.329), anti-SSA (56.0 vs 57.3%, *P* = 0.907), and anti-SSB (40.0 vs 37.3%, *P* = 0.812) was not different between the non-survivors and survivors, suggesting that autoantibodies were not associated with prognosis of ILD^44^. However, there is also a contradictory report; Boitiaux et al., in 45 newly diagnosed patients with idiopathic interstitial pneumonia, showed that the anti-SSA (+) group (n = 15) had lower vital capacity (63 ± 22 vs. 87 ± 23% predicted, *P* = 0.006) and more frequent GGO (87 vs 67%, *P* = 0.001) and reticulation (33 vs 21%, *P* = 0.030) on HRCT than the anti-SSA(−) group (n = 30)^45^. Due to these inconsistent findings, it is still insufficient to draw a conclusion about the association between autoantibodies and prognosis of SJS-ILD.

Our study has some limitations. First, this was a retrospective study conducted at a single center, which might lead to selection biases or a lack in generalizability. However, the baseline characteristics of our patients were comparable to those in other studies^12,17^. Second, we did not include a validation cohort to confirm our findings. Our sample size was relatively small; however, the rarity of SJS-ILD means that the number of patients studied is not considered low, and the long-term observation in our study can provide important insights into this rare condition. Finally, detailed treatment information such as type, dose, timing, and the duration over which medication was given were not considered in the analysis of the prognostic factors. However, the treatment given to survivors and non-survivors did not differ.

In conclusion, our results suggest that blood KL-6 might be a useful predictor of the prognosis for patients with SJS-ILD.

## Supplementary Information

Supplementary Information.

## Data Availability

The datasets generated during and/or analyzed during the current study are available from the corresponding author on reasonable request.

## Abbreviations

6MWD: Six-minute walk test distance
6MWT: Six-minute walk test
AE: Acute exacerbation
ATS: American Thoracic Society
AUC: Area under a curve
BAL: Bronchoalveolar lavage
CCL18: CC chemokine ligand 18
CI: Confidence interval
CTD: Connective tissue disease
DL_CO_: Diffusing capacity of the lung for carbon monoxide
ERS: European Respiratory Society
ESSDAI: European League Against Rheumatism Sjögren’s Syndrome Disease Activity Index
FVC: Forced vital capacity
GGO: Ground glass opacities
HR: Hazard ratio
HRCT: High resolution computed tomography
IL-4Ra: Interleukin-4 receptor alpha
ILD: Interstitial lung disease
IPF: Idiopathic pulmonary fibrosis
IQR: Interquartile range
KL-6: Krebs von den Lungen-6
MMP-7: Matrix metalloproteinase-7
OR: Odds ratio
RA: Rheumatoid arthritis
ROC: Receiver operating characteristic
SJS: Sjögren syndrome
TLC: Total lung capacity
UIP: Usual interstitial pneumonia
YKL 40: Chitinase-3-like
