# Supplementary material for: Blood KL-6 predicts prognosis in primary Sjögren’s syndrome-associated interstitial lung disease
Source: Sci Rep. 2022 Mar 29;12:5343. doi: 10.1038/s41598-022-09283-w (PMC8964755; doi:10.1038/s41598-022-09283-w)
Supplement: Supplementary file 1 — Supplementary Information. [file 41598_2022_9283_MOESM1_ESM.doc]

**Blood KL-6 predicts prognosis in primary Sjögren’s syndrome-associated interstitial lung disease**

Yun Jae Kim^1^, Jooae Choe^2^, Su-Jin Moon^3^, Jin Woo Song^3*^

^1^University of Ulsan College of Medicine, Seoul, Republic of Korea

^2^Department of Radiology and Research Institute of Radiology, University of Ulsan College of Medicine, Asan Medical Center, Seoul, Republic of Korea.

^3^Department of Pulmonary and Critical Care Medicine, Asan Medical Center, University of Ulsan College of Medicine, Seoul, Republic of Korea

**Supplementary Table S1.** Comparison of baseline characteristics between the patients who were included and excluded in this study from the cohort

| Characteristics | Patients excluded | Patients included | *P* value |
| --- | --- | --- | --- |
| Patient number | 16 | 46 |  |
| Age, years | 60.8 ± 13.9 | 59.4 ± 10.6 | 0.677 |
| Male | 2 (12.5) | 8 (17.4) | 1.000 |
| Smoking history | 2 (12.5) | 12 (26.1) | 0.322 |
| ANA, positive (>1:40) | 13 (81.3) | 34 (73.9) | 0.739 |
| Anti SS-A/Ro, positive | 15 (93.8) | 33 (71.7) | 0.090 |
| Anti-SS-B/La, positive | 9 (56.3) | 16 (34.8) | 0.151 |
| C-reactive protein, mg/dL | 0.2 ± 0.2 | 1.4 ± 2.9 | 0.012 |
| UIP pattern on HRCT | 6 (37.5) | 18 (39.1) | 1.000 |
| FVC, predicted % | 71.8 ± 16.7 | 67 ± 13.9 | 0.275 |
| DL_CO_, predicted % | 62.5 ± 20.3 | 57.7 ± 18.4 | 0.415 |
| TLC, predicted % | 73.6 ± 15.7 | 70.7 ± 13.7 | 0.515 |
| 6MWD, meters | 406.3 ± 133.5 | 425.1 ± 107.1 | 0.617 |
| 6MWT the lowest SpO_2_, % | 93.7 ± 4.1 | 91.2 ± 4.3 | 0.080 |
| BAL neutrophil, % | 12.6 ± 16.4 | 12.4 ± 18.2 | 0.984 |
| BAL lymphocyte, % | 24.4 ± 14.4 | 27.2 ± 15.8 | 0.653 |

Data are presented as mean ± standard deviation or number (%), unless otherwise indicated. 6MWD, six-minute walk test distance; 6MWT the lowest SpO2, lowest oxygen saturation during the six-minute walking test; ANA, anti-nuclear antibody; BAL, bronchoalveolar lavage; DL_CO_, diffusing capacity of the lung for carbon monoxide; FVC, forced vital capacity; HRCT, high resolution computed tomography; ILD, interstitial lung disease; SJS, Sjögren syndrome; TLC, total lung capacity; UIP, usual interstitial pneumonia.

**Supplementary Table S2.** Causes of death in patients with SJS-ILD

| Characteristics | Non-survivors |
| --- | --- |
| Patient number | 12 |
| Underlying ILD progression | 8 (66.7) |
| Pneumonia | 1 (8.3) |
| Tuberculosis | 1 (8.3) |
| Heart failure due to hypertensive heart disease | 1 (8.3) |
| Unknown | 1 (8.3) |

Data are presented as number (%).

SJS-ILD, Sjögren’s syndrome-associated interstitial lung disease
